# Subject Combination and Electrode Selection in Cooperative Brain-Computer Interface Based on Event Related Potentials

**DOI:** 10.3390/brainsci4020335

**Published:** 2014-04-30

**Authors:** Hubert Cecotti, Bertrand Rivet

**Affiliations:** 1 School of Computing and Intelligent Systems, University of Ulster, Derry BT48 7JL, Northern Ireland, UK; 2 GIPSA-lab CNRS UMR 5216, Grenoble Universities, Saint Martin d’Heres 38400, France; E-Mail: bertrand.rivet@gipsa-lab.grenoble-inp.fr

**Keywords:** Brain-Computer Interface, cooperative mode, event-related potentials (ERP), electrode selection

## Abstract

New paradigms are required in Brain-Computer Interface (BCI) systems for the needs and expectations of healthy people. To solve this issue, we explore the emerging field of cooperative BCIs, which involves several users in a single BCI system. Contrary to classical BCIs that are dependent on the unique subject’s will, cooperative BCIs are used for problem solving tasks where several people shall be engaged by sharing a common goal. Similarly as combining trials over time improves performance, combining trials across subjects can significantly improve performance compared with when only a single user is involved. Yet, cooperative BCIs may only be used in particular settings, and new paradigms must be proposed to efficiently use this approach. The possible benefits of using several subjects are addressed, and compared with current single-subject BCI paradigms. To show the advantages of a cooperative BCI, we evaluate the performance of combining decisions across subjects with data from an event-related potentials (ERP) based experiment where each subject observed the same sequence of visual stimuli. Furthermore, we show that it is possible to achieve a mean AUC superior to 0.95 with 10 subjects and 3 electrodes on each subject, or with 4 subjects and 6 electrodes on each subject. Several emerging challenges and possible applications are proposed to highlight how cooperative BCIs could be efficiently used with current technologies and leverage BCI applications.

## 1. Introduction

The field of non-invasive Brain-Computer Interface (BCI) has been particularly active during the last two decades thanks to pioneering works such as the P300 speller [1], which opened the path to a new research field combining neuroscience, signal processing, machine learning and human-computer interaction. Over these years, BCIs have mainly been used as a way to control a computer and to use different type of devices. In all these contexts, the interaction between the subject and the devices has always been in relation to the subject’s will. The paradigms have largely been related to communication purposes: to send commands to a computer, e.g., spelling words [2], and in assistive technologies [3]. Due to the low signal-to-noise ratio (SNR) of the electroencephalogram (EEG) signal, many strategies have been proposed to increase the SNR, e.g., the combination of several trials over time thanks to the repetition of different events [4]. In the rest of the paper, we distinguish trials from events. A trial corresponds to the EEG signal recorded during an event, which is time locked. Several trials can be recorded for the same event, *i.e.*, from several subjects; several trials can be recorded for different events that have the same meaning, *e.g.*, the presentation at different times of the same item in a P300 speller.

Whereas combining EEG signals over time has been used since the first P300 speller [1], where the stimuli appearing on the items of the speller layout are repeated several times, combining EEG signals across subjects in BCI has not been efficiently used until recently for decreasing reaction time [5,6], and for 2-D pointer control with two users [7]. In this type of BCI, several users are implicated for enabling a command. In [8], the activity of multiple brains was analyzed to reveal causal connections between regions of different brains (hyperconnectivity). In [9], it was suggested that multi-brain computing can be used as a technique to rapidly, in *parallel*, gather increased information about the environment, and to access collective perceptual/cognitive choices and mental states. In [10,11], the interest of combining behavioral responses and neural features was shown as a means to improve accuracy when compared with individual performance.

Although it seems that combining EEG signals across subjects is identical to the combination of EEG signals over time at the signal processing level, we show that cooperative BCIs need specific paradigms, and should be used only in particular conditions. By considering a state-of-the-art experiment with additional constraints due to the cooperative feature, we address in this study two key questions: (i) what is the best combination method for combining decisions across subjects; and (ii) to what extent is there a difference of performance between considering as inputs a large set of electrodes on a single subject and a small set of electrodes with several subjects.

The remainder of this paper is organized as follows. First, we review in Section 2 some current issues and bottlenecks in BCI that limit commercial applications. In Section 3, we define the notion of cooperative BCI, and its difference compared with classical BCI paradigms. Then, the performance obtained by combining signals across subjects is presented for the detection of event-related potentials (ERPs). This experiment is used to determine the best trade-off between the number of subjects and the number of electrodes common to each subject to optimize the overall performance, *i.e.*, is it better to consider a single subject wearing an EEG cap with eight electrodes, or four subjects with each one having only two electrodes? Finally, the future challenges and possibilities of cooperative BCI are detailed in Section 6.

## 2. BCI Issues

In spite of all the recent advances in non-invasive BCI, there remain a long list of issues that prevent BCIs from being used efficiently by healthy people. In order to improve the reliability of BCI, several paths have been explored. First, new signal processing and machine learning techniques have been proposed to increase the performance of detecting brain responses. These methods currently address the challenges related to the non-stationary characteristics of the EEG signal and the high variability that can happen over time and across subjects [12,13,14]. For instance, the performance of the P300 speller can be increased by using intersubject information and online adaptation [15], by sensor selection [16,17], and by decreasing the number of repetitions [18]. Despite all such improvements, the accuracy of single-trial detection remains far from sufficient. In addition, improvements can come from an optimization of the BCI in relation to the application, e.g., word completion and prediction for virtual keyboards [19]. Second, to face the obstacles related to the poor performance, BCIs have been combined with other communication devices or multiple BCIs. This idea was introduced as the hybrid BCI concept [20,21]. In hybrid BCIs, it is important to distinguish pure hybrid BCIs where the control remains fully BCI dependent and other hybrid systems that embed other physiological signals or communication devices.

Moreover, commercial non-invasive BCI remains limited to a general audience due to the lack of efficient systems with dry electrodes [22,23]. It is, in fact, difficult to obtain a good signal on the hairy parts of the head. This is why new studies explore the effect of electrodes on non-hair-bearing areas [24]. Companies such as NeuroSky, Neuroelectrics, Quasar [25], G.tec with g.Sahara, and Mindo from the Brain Research Center (BRC) of the National Chiao Tung University, Taiwan [26], have proposed portable solutions with dry electrodes for recording EEG signals. These efforts show that issues related to the uncomfortable aspect of BCI tend to be solved in the near future and the remaining issue would become the BCI performance only (speed and accuracy).

## 3. Cooperative BCI

### 3.1. Extending the Notion of BCI

**Figure 1:**
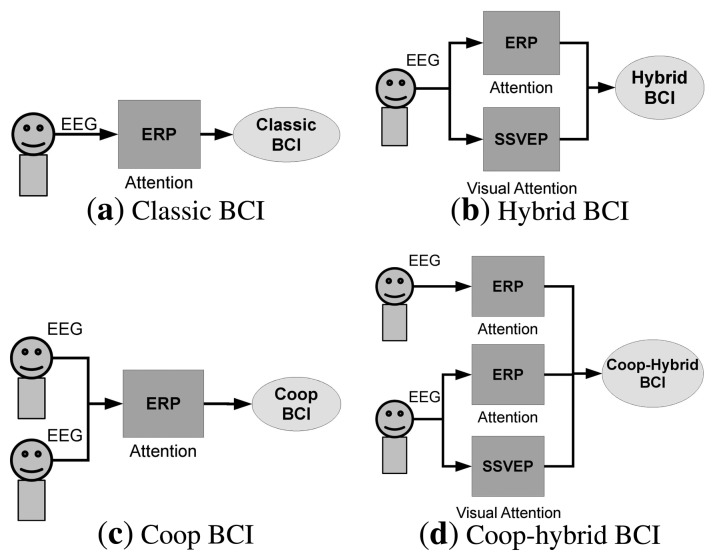
Examples of different BCI types.

While hybrid BCI [21] has extended the concept of BCI, we propose here to classify a BCI according to the number of brain response types that are involved in the system and to the number of people that are involved in the task. Hence, four types of BCI can be distinguished:

1. The *classical* BCI: it involves a single person and a single brain modality, *i.e.*, a single type of brain response (ERP or SSVEP, motor imagery, …) (see Figure 1a).
2. The *hybrid* BCI: it involves a single person, and the possibility to consider several brain modalities (see Figure 1b).
3. The *cooperative* BCI: it involves a group of people, and a single brain modality (see Figure 1c).
4. The *cooperative-hybrid* BCI: it involves a group of people, and the possibility to consider several brain modalities. The type of brain response can depend on the subject, *i.e.*, the BCI can be hybrid only for some subjects (see Figure 1d).

In current BCIs, the subject typically interacts with the computer by sending commands through the detection of brain responses. Those commands correspond to the user’s will: the subject is the only person controlling the application. In a cooperative BCI, the decision is based on the combination of brain signals from multiple users [27,28]. Cooperative BCIs can be seen as particular types of pure hybrid BCIs. Instead of having a system accepting inputs from a single subject and several types of signals (e.g., P300 and SSVEP) (see Figure 1b), a cooperative system (hybrid or not) accepts inputs from several subjects and one or several types of signals (see Figure 1c,d). In this sense, the BCI accepts different inputs coming from different subjects. Contrary to pure hybrid BCIs, where the user may have to pay attention to several types of stimuli, *i.e.*, a flickering light for SSVEP, and an oddball paradigm for ERPs, each subject can focus his attention on a single type of stimulus in a cooperative BCI. Since differences exist between cooperative BCIs and current BCIs, we would like to distinguish single-trial and single-event detection. In single-trial detection, the response is detected with only one trial, whereas in single-event detection the event can be represented by a series of trials that were recorded across several subjects.

**Figure 2:**
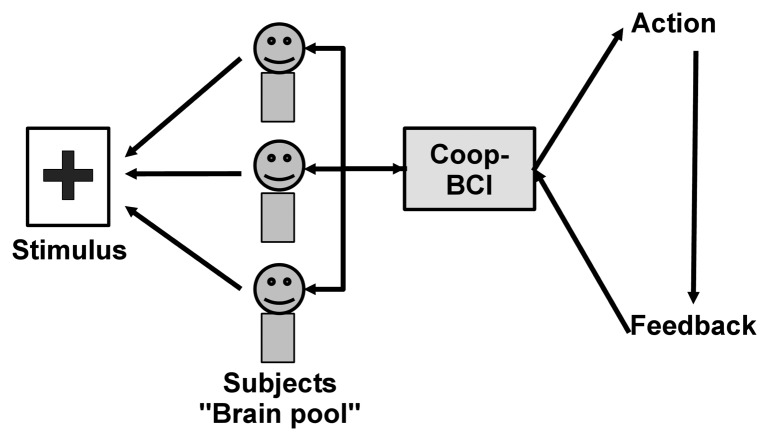
Cooperative BCI based on the detection of ERPs.

It is worth noting that cooperative BCIs cannot be used for applications depending only on the subject’s will. For instance, for virtual keyboard applications, the selection of the letters that are written depends only on the person who is writing the text (it is possible to do copy spelling in cooperative mode but it is not the main goal of a BCI virtual keyboard). A typical scheme of a cooperative BCI based on the detection of event-related potential is presented in Figure 2. In this example, a pool of subjects shall pay attention to a stimulus and the detection of this stimulus enables a command, which provides a feedback to the pool of subjects. In order to have all the subjects paying simultaneous attention to the stimulus, an adapted task must be chosen. Yet, this situation can happen in the case of an emergency signal where an event triggers a particular response across all subjects.

### 3.2. Combination Strategies

The literature of multi-classifier systems offers a large choice of methods for combining the signals and/or the decisions from different subjects [29,30]. Increasing the number of subjects can have different impact on the performance. By adding more subjects, it is possible to add subjects who have had bad performances: although their individual performance does not allow them to use a BCI as a single user, their brain features can still somewhat contribute to the global performance. The choice of the combination strategy may depend on how possible it is to find subjects that can complement each other, particularly for adaptive combination methods. In this study, we limit the applications to strategies for binary classification.

Let *O_i_* denote the output value of a binary classifier for subject *i* at a particular trial. *O_i_* (0 ≤ *O_i_* ≤ 1) represents the confidence value of assigning the input to one of the two classes. Six classical combination scores over *N_sub_* subjects are considered:

1. The sum of the classifier outputs (*O_sum_*):
  (1) $$O_{\text{sum}}=\sum_{i=1}^{N_{\text{sub}}}O_{i}$$
2. The weighted sum of the classifier outputs (*O_w_sum_*):
  (2) $$O_{w-\text{sum}}=w_{0}+\sum_{i=1}^{N_{\text{sub}}}w_{i}\cdot O_{i}$$
3. The product of classifier outputs (*O_prod_*):
  (3) $$O_{\text{proud}}=\prod_{i=1}^{N_{\text{sub}}}O_{i}$$
4. The selection of the maximum score (*O_max_*):
  (4) $$O_{\text{max}}=\underset{1\leq i\leq N_{\text{sub}}}{\max}O_{i}$$
5. The selection of the minimum score (*O_min_*):
  (5) $$O_{\text{min}}=\underset{1\leq i\leq_{\text{sub}}}{\min}O_{i}$$
6. The voting (*O_v_*(*k*)):
  (6) $$Ov\left(k\right)=\{\begin{array}{ll} 1, & \text{if}\sum_{i=1}^{N_{\text{sub}}}D_{i}\left(\alpha_{i}\right)\geq k\\ 0, & \text{otherwise} \end{array}$$
  where *D_i_*(*α*) is the binary decision associated with the *i*-th subject defined by
  (7) $$D_{i}\left(\alpha \right)=\{\begin{array}{ll} 1, & \text{if}O_{i}>\alpha \\ 0, & \text{otherwise} \end{array}$$
  and *α* is a threshold for the decision to assign the input signal to the target or non-target class. Two particular cases of voting can be distinguished: the majority vote where *k* = *N_sub_*/2, and the consensus vote where *k* = *N_sub_*, which are denoted *O_maj_* and *O_cons_*, respectively.

It is worth mentioning that these scores are related to different intuitive strategies. Hence, the maximum score (*O_max_*) and the minimum score (*O_min_*) are related to the notions of OR and AND in logic, respectively. The maximum score makes its decision based on the most relevant subject (*i.e.*, with the highest individual score *O_i_*): a single relevant subject is sufficient to provide a high combined score. On the contrary, the minimum score makes its decision based on the least relevant subject (*i.e.*, with the lowest individual score *O_i_*): all the subjects have to be relevant to provide a high combined score. Moreover, the sum (*O_sum_*) and weighted sum (*O_w_*_–_*_sum_*) scores can be seen as smooth or weighted OR, while the product score (*O_prod_*) is a smooth or weighted AND.

The choice of the combined score depends not only on these considerations but also on the distribution of the estimation errors. Kittler *et al.* [31] showed that for Gaussian distributions it is better to use the sum (or mean) strategy than majority vote (*i.e.*, *O_v_*(*k*) with *k* = *N_sub_*/2). Yet, for heavy tail distributions, the majority vote can give better results than the sum.

### 3.3. Performance Evaluation

To show the relevance and the type of performance that can be expected by combining the decision from several individuals, we consider data obtained from a P300 speller experiment where the goal is to detect ERPs. The performance will be reported with the Area Under the ROC curve (AUC) [32] and the information transfer rate (ITR) [33] in bits per minute (bpm) defined by:

(8) $$\text{ITR}=\frac{60}{T}\cdot \vartheta$$

where *ϑ* is the capacity of the channel between the interface and the user’s brain:

(9) $$\vartheta =\log_{2}\left(N_{\text{out}}\right)+P\log_{2}\left(P\right)+\left(1-P\right)\log_{2}\left(\frac{1-P}{N_{\text{out}}-1}\right)$$

with *P* being the probability of a good detection (*i.e.*, the accuracy), *N_out_* being the number of possible different outputs, and *T* being the time in seconds of recorded EEG signal that is required to make the decision among the *N_out_* outputs.

In addition, the ITR of a cooperative BCI can be normalized to take into account the number of people involved in the decision, as the overall performance depends on the number of people involved in the task, the performance of the detection, and the time to perform the task. We define the normalized ITR by:

(10) $${\text{ITRP}}_{f}=\frac{60}{T\cdot f\left(N_{\text{sub}}\right)}\cdot \vartheta$$

where *f*(*N_sub_*) is a cost function that takes as argument the number of subjects, and *f*(l) = 1. As amplifiers have typically a number of electrodes that is a power of 2, we consider *f*(*N_sub_*) = 1 + *log*_2_(*N_sub_*).

Since the performance of a BCI depends on the number of electrodes, and the number of electrodes largely determines the cost of a BCI system, the number of electrodes becomes a pertinent parameter for the performance estimation.

## 4. Single-Event ERP Detection

### 4.1. Participants and Study Design

To evaluate the effect of combining several subjects, we considered classifier outputs from ten healthy subjects (age = 25.5 ± 4.4 years old, three females) that were acquired during P300 speller experiments. Although the P300 speller is not the typical cooperative BCI application, the P300 experiment represents the most common paradigm in BCI based on ERP detection, and it has become a type of benchmark experiment for evaluating new BCI approaches. In addition, there may be applications that would require several people to spell the same word. Each subject had to spell a total of 40 characters, and the experiments were carried out sequentially. Because subjects could be at different location and the final results may be transferred to a different person, the sequential experiment was not an issue for simulating the performance of a cooperative BCI. The most important characteristic of the experiments was that the characters to spell and the order of the stimuli were identical across subjects. Hence, each subject observed the same sequence of visual stimuli. The matrix of the P300 speller was 6 × 6 and displayed on a 27 inch LCD screen with a brightness of 375 cd/m^2^. Subjects sat on a chair at about 60 cm from the screen in a non-shielded room. The stimulus onset asynchrony was set to 133 ms and the inter-stimulus interval was 66 ms, the signal was recorded on *O*_1_, *O*_2_, *P*_3_, *p*_4_, *p*_7_, *p*_8_, *p_z_* and *FC_z_*. *F*7 and *F*8 were dedicated to the ground and the reference, respectively. The amplifier was a FirstAmp (Brain Products GmbH) with a sampling frequency of 2 kHz.

### 4.2. Signal Processing and Classification

The signal was first bandpass filtered (Butterworth filter of order 4) with cutoff frequencies at 1 and 12.5 Hz, then downsampled to 25 Hz. The next processing step was spatial filtering with the xDAWN method, which maximizes the signal to signal-plus-noise ratio [34,35]. The technique has been successfully applied in P300-based BCI and in RSVP tasks [16,36]. Let us denote the spatial filters by *U* ∈ ℝ*^Ns^*^×^*^Nf^*, where *N_f_* is the number of spatial filters. The signal after spatial filtering is defined by *X_filt_* = *XU*, where *X* ∈ ℝ*^Nt^*^×^*^Ns^* is the recorded signal, *N_t_* is the number of sampling points, and *N_s_* is the number of electrodes. An algebraic model of the enhanced signals *XU* is composed of three terms: the ERP responses on a target class (*D*_1_*A*_1_), a response common to all stimuli, *i.e.*, all targets and non-targets confound (*D*_2_*A*_2_) and the residual noise (*H*, a real matrix of size *N_t_* × *N_s_*), which are filtered spatially with *U*.

(11) $$XU=\left(D_{1}A_{1}+D_{2}A_{2}+H\right)U$$

where *D*_1_ and *D*_2_ are two real Toeplitz matrices of size *N_t_* × *N*_1_ and *N_t_* x *N*_2_, respectively. *D*_1_ has its first column elements set to zero except for those that correspond to a target onset, which are represented with a value equal to one. For *D*_2_, its first column elements are set to zero except for those that correspond to stimuli onset. *N*_1_ and *N*_2_ are the number of sampling points representing the target and superimposed evoked potentials, respectively. *A*_1_ ∈ ℝ*^N^*^1×^*^Ns^* and *A*_2_ ∈ ℝ*^N^*^2 ×^ *^Ns^* represent the ERP response on a target class and the ERP response on every stimulus, respectively. The spatial filters *U* maximize the signal to signal-plus-noise ratio (SSNR):

(12) $$\text{SSNR}\left(U\right)=\arg\max_{U}\frac{Tr\left(U^{T}{\hat{A}}_{1}^{T}D_{1}^{T}D_{1}{\hat{A}}_{1}U\right)}{Tr\left(U^{T}X^{T}XU\right)}$$

where *Â*_1_ corresponds to the least mean square estimation of *A*_1_.

(13) $$\hat{A}=\left[\begin{array}{c} {\hat{A}}_{1}\\ {\hat{A}}_{2} \end{array}\right]$$

(14) $$A={\left({\left[D_{1};D_{2}\right]}^{T}\left[D_{1};D_{2}\right]\right)}^{-1}{\left[D_{1};D_{2}\right]}^{T}X$$

where [*D*_1_;*D*_2_] is a matrix of size *N_t_* × (*N*_1_ + *N*_2_) obtained by concatenation of *D*_1_ and *D*_2_. Spatial filters are obtained through the Rayleigh quotient by maximizing the SSNR [34]. During the experiments, four spatial filters (*N_f_* = 4) were used. The input vector for the classifier was obtained by the concatenation of the *N_f_* time-course signals across spatial filiters for a single subject. The detection score, *O_i_*, was obtained with the Bayesian linear discriminant analysis (BLDA) classifier [37]. The training and test database contained 4800 trials for each subject (800 for target, 4000 for non-target). We evaluated the impact of the number of subjects on single-event detection by considering the classifier outputs from the different subjects with Monte-Carlo simulations (combination of trials corresponding to same events, e.g., the presentation of a visual stimulus, and combination of trials from different subsets of subjects).

### 4.3. Results

**Figure 3:**
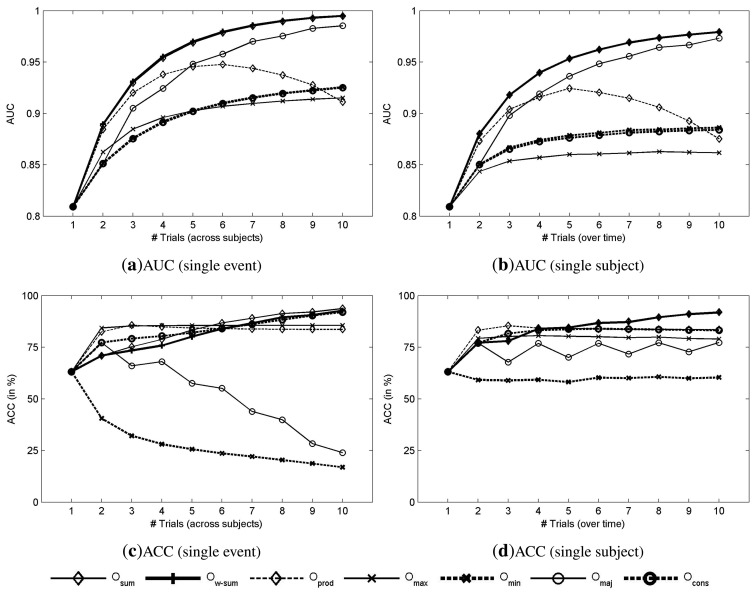
Detection results based on the AUC and the accuracy (ACC). (**a,b**) AUC for single-event detection as a function of the number of subjects, and AUC for a single subject as a function of the number of trials, respectively. (**c,d**) Accuracy for single-event detection as a function of the number of subjects, and accuracy for a single subject as a function of the number of trials, respectively.

**Figure 4:**
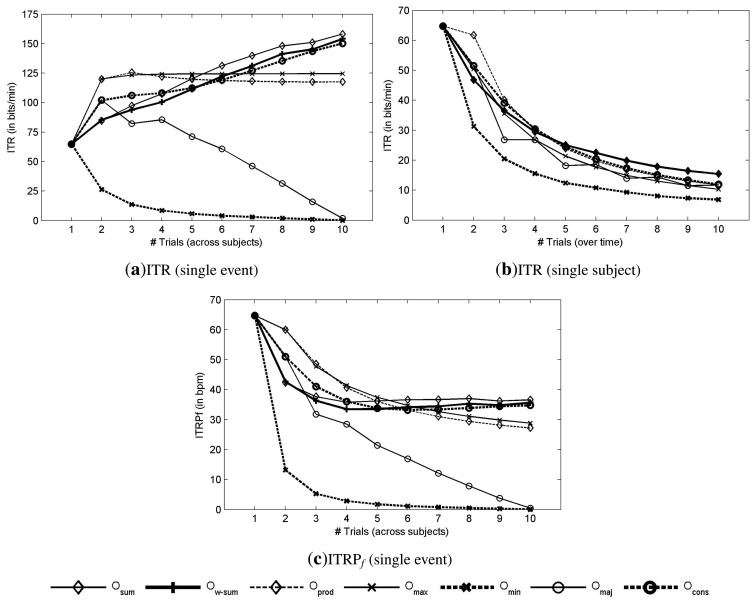
Detection results based on the ITR and ITRP*_f_*. (**a,b**) theoretical ITR as a function of the number of subjects and the number of trials, respectively. (**c**) theoretical ITRP*_f_* as a function of the number of subjects and the number of trials, respectively.

For single-event detection, the mean AUC across subjects is 0.810 ± 0.067. The results for single-trial detection are depicted in Figure 3 and Figure 4; they represent the mean AUC, accuracy, ITR, and ITRP*_f_* across all the possible combinations of *n* subjects (Figure 3a,c and Figure 4a,c), and across all the possible combinations of *n* trials from the same subject (Figure 3b,d and Figure 4b).

The results presented in Figure 3a confirm that aggregating subjects can improve the classification accuracy estimated by AUC. For instance, with the sum combined score (*O_sum_*) the AUC increases from 0.810 ± 0.061 with a single subject to 0.995 ± 0.000 with ten subjects. However, as expected, the AUC achieved with the product (*O_prod_*) and minimum (*O_min_*) scores do not monotonically increase with the number of subjects: there is a maximum AUC at about five subjects. Consequently, as already noted, these combined scores require that all the individual scores be relatively high to achieve a good detection while increasing the number of subjects reduces this probability.

The evolution of AUC as a function of the number of subjects involved in the decision (Figure 3a) and as a function of the number of trials combined over time (Figure 3b) presents the same behavior depending on the number of combined scores. The accuracy is depicted in Figure 3c,d. In both cases, the accuracy starts at about 63.17% ± 16.29% with a single-trial, and increases to about 85% with only two trials, then it reaches a plateau at 90%. Finally, the accuracy becomes 93.72% ± 1.69% with *O_sum_* and 92.74% ± 3.20% for *O_w_*_–_*_sum_* with ten subjects.

Figure 4a shows the theoretical ITR (8) as a function of the number of subjects that are involved in the decision. The ITR was estimated based on the choice among six commands (*N_out_* = 6), because of the matrix size and a time of 0.133 • 6 *s* for the selection of a command (*T* = 0.8 *s*). The best performance is obtained with the sum method (*O_sum_*) with an ITR of 158.14 ± 7.83 bpm with 10 subjects. For *O_sum_*, the ITRP*_f_* decreases with the addition of subjects to reach 36.59 bpm with 10 subjects, as depicted in Figure 4c. As expected from the AUC plots, when the AUC increases with the number of involved subjects, the ITR also increases. Contrary to combination over trials (with a single subject), which increases the time to make a decision, which is defined by *T* = 0.8 * #*trials*, this time is exactly the same when combining over subjects with a single trial for each. As a consequence, the increase of AUC is directly reflected on the ITR (Figure 4a), while it is not the case when combining over trials (Figure 4b). One can compare the aggregation of trials with sequential processes while the aggregation of subjects is more related to parallel processes. The evolution of the performance indicates a continuous increase of performance with the addition of new subjects. With about four or five subjects, it is possible to achieve a performance that is reliable enough for single-event detection.

## 5. Electrode Selection in Cooperative BCIs

The rationale behind considering several subjects is the same as considering several electrodes: the goal is to increase the number of information channels that are used in the decision. Those information channels can come from different locations on the head and/or different subjects. In this section, we propose to compare the performance obtained with different number of electrodes and different number of subjects. We evaluate the best electrode subsets from size 1 to *N_s_* that maximizes the mean SSNR across subjects. Here, SSNR is estimated after the xDAWN spatial filtering method. Hence, the score function is estimated globally as a function of the mean grouped SSNR from the virtual electrodes. We consider two approaches for electrode selection. In the first approach (*SS_individual_*), we determine the electrode subsets for each individual, as described in [16], hence *N_sub_* electrode subsets are created. In the second approach (*SS_common_*), electrode selection optimizes the mean SSNR across subjects and not the SSNR of each subject, because EEG systems with a low number of electrodes are typically fixed, and it is not possible to adjust the location of the electrodes. With *SS_common_*, a single electrode subset is created.

**Figure 5:**
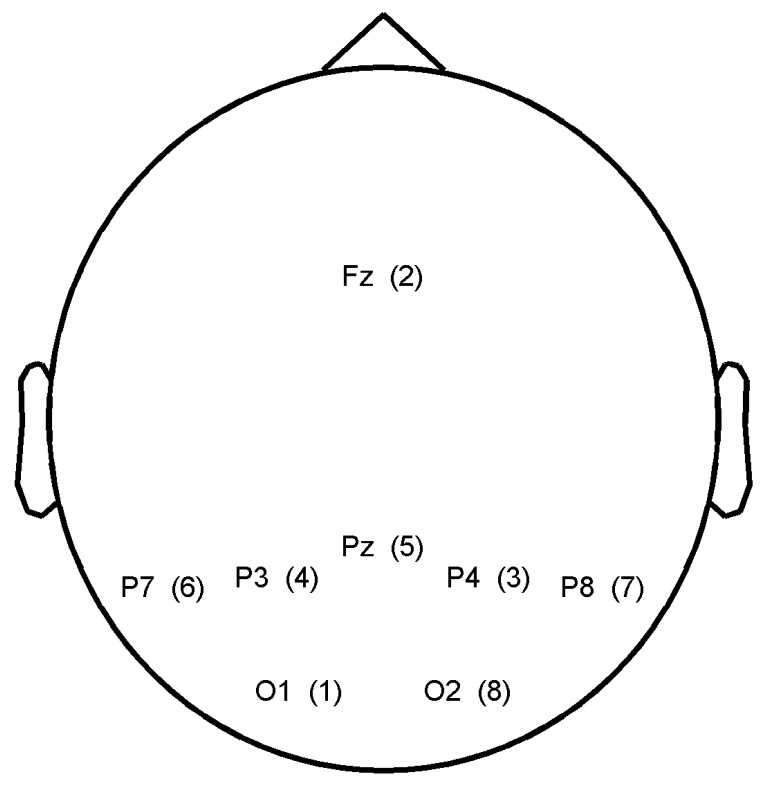
Electrode scores after electrode selection based on the maximization of the mean SSNR across subjects (*SS_common_*).

**Figure 6:**
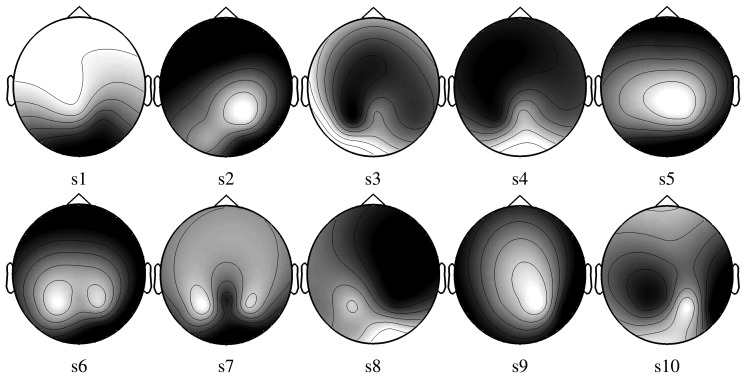
Electrode scores after electrode selection based on the maximization of the SSNR for each subject (*SS_individual_*). (A low gray level represents a high score for the electrode.)

In both approaches, the electrode selection procedure is based on a recursive backward elimination. Starting with all electrodes, it consists in testing each electrode for its significance across subjects and in removing the least relevant one at each iteration step. An irrelevant electrode is an electrode whose removal barely impairs the performance or selection criterion. Elimination goes on until every electrode has been eliminated. The score corresponding to the subset selection for *SS_cornrnon_* is given for each electrode between brackets in Figure 5. The best electrodes are *O*_2_ and *P*_8_. The electrode selected for each subject with *SS_individual_* are depicted in Figure 6.

The electrode subsets are estimated with data from the first session, which contain 400 patterns corresponding to the presentation of a target and 2000 patterns for the presentation of a non-target. The remaining data are considered for evaluating classifier performance (AUC). We define the AUC performance *auc*(*i*, *j*) by the AUC obtained by considering *i* electrodes, 1 ≤ *i* ≤ *N_s_* and *j* subjects, 1 ≤ *J* ≤ *N_sub_*. *auc*(*i*, *j*) is computed by considering the mean AUC with the *O_sum_* rule of all the possible combinations of *j* subjects (*N_sub_*!*/*((*N_sub_* − *j*)! · *j*!), where! is the factorial function).

**Figure 7:**
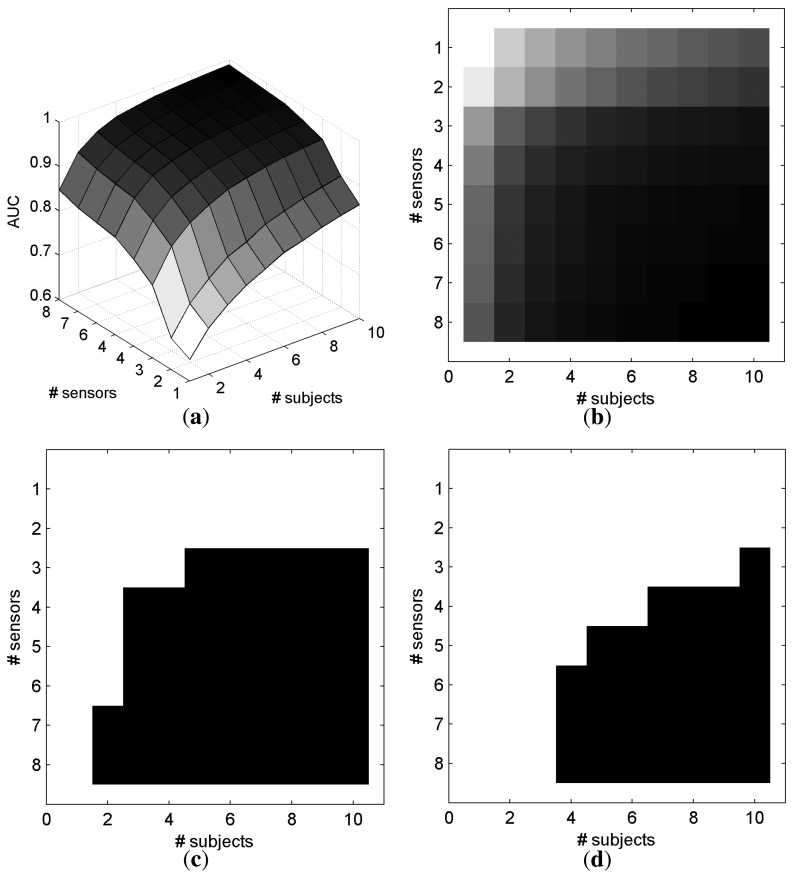
AUC based on the number of electrodes and subjects with the *SS_common_* method for electrode selection and *O_sum_* for fusing the classifier outputs across subjects. (**a**) 3D representation of the AUC; (**b**) 2D representation of the AUC; (**c**) *AUC* > 0.90; (**d**) *AUC* > 0.95. (In Figure 7b, a dark gray level represents a high AUC.)

**Figure 8:**
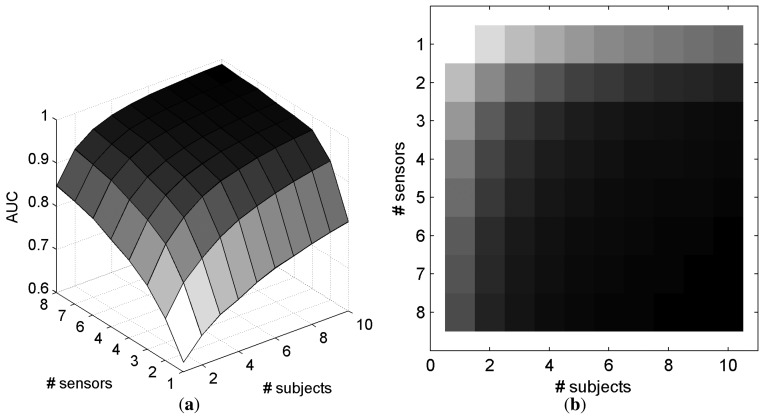
AUC based on the number of electrodes and subjects with the *SS_individual_* method for electrode selection and *O_sum_* for fusing the classifier outputs across subjects. (**a**) 3D representation of the AUC; (**b**) 2D representation of the AUC. (In Figure 8b, a dark gray level represents a high AUC.)

Figure 7 represents the AUC as a function of the number of electrodes and number of subjects that are involved in the classification for *SS_common_*. With only one electrode and one subject, the mean AUC is 0.649. The maximum AUC (0.989) is obtained with the maximum information, *i.e.*, eight electrodes and ten subjects. For a better understanding, Figure 7c,d depicts in black when a configuration of electrodes and subjects allows an AUC superior to a defined threshold for *SS_common_*. Particularly, it is possible to achieve a mean AUC superior to 0.95 with 10 subjects and 3 electrodes on each subject, or with 4 subjects and 6 electrodes on each subject. Similar results were observed with electrode selection obtained with *SS_individual_*, as depicted in Figure 8.

For a constant number of electrodes involved in the decision, we have compared the AUC between the configurations where the number of electrodes in a single subject is greater than the number of subjects and vice versa. We define a configuration of subjects and electrodes by *C*(*i*, *j*), where *i* is the number of subjects, and *j* is the number of electrodes. For instance, we compared the performance of having one subject with eight electrodes against having eight subjects and one electrode, and having four subjects each with two electrodes. Hence, we obtained 56 configurations (*C*(1, 2)*vs.C*(2,1), *C*(1, 8)*vs.C*(2,4), *C*(1, 8) *vs. C*(4, 2), *C*(1, 8) *vs. C*(8,1),…). The analysis of all the possible combinations resulted into 44 pairwise comparisons with the same global number of electrodes.

With *SS_common_*, a pairwise *t*-test comparison revealed that the configurations with more electrodes in a single subject, than subjects involved in the decision, provided better performance (*t*_43_ = 3.52, *p* = 0.001). These results suggest that it is better to consider smaller number of subjects with more electrodes than otherwise. The same pattern of performance was observed with *SS_individual_*, (*t*_43_ = 4.10, *p* < 1.76*e* − 4). However, there exist 12 and 17 configurations (for *SS_common_* and *SS_individual_*, respectively), where the performance is higher while considering a higher number of subjects compared with the number of electrodes per subject.

## 6. Discussion

Cooperative BCIs involve the creation of new systems and new ways of thinking in terms of BCI systems. We have shown that it is possible to significantly increase the ITR with simple combination methods by considering the detection of event-related potentials occurring simultaneously across subjects. However, a key constraint is the number of subjects to involve in the decision. The trade-off between the time needed to perform a task and the number of subjects involved in the decision should be chosen in relation to the application. A key result is the difference in performance that can occur between a classical BCI and a cooperative BCI for the detection of two brain responses.

Furthermore, it is difficult to compare a single-user BCI and a cooperative BCI involving several users because the goal of both systems can be different, but it is still possible to evaluate the accuracy and the speed to produce commands. In particular, when it comes to applications, it is not relevant to compare the mean performance across subjects for a single BCI user with trials combined over time against the performance of a cooperative BCI with trials combined over subjects, because these applications have different purposes. Yet, there are exceptions such as when a group of subjects should write the same sentence with a BCI speller. Like other BCI experiments, some limitations of the study include the effects that are related to the motivation and the interest of the subjects in the experiment.

The results suggest that differences between two types of brain responses that could not be efficiently used for single-user BCI application may be advantageously useful in cooperative BCIs. Particularly, ERP components that require a large number of subjects to be detected could be used in a cooperative BCI system. In fact, ERP components such as the P300 and N200 are used in BCI because their amplitudes allow them to be relatively easy to detect at the single-trial detection level. Yet, particular ERP features (amplitude and latency) are used in psychology to model differences across experimental conditions [38]. These features could be considered in a cooperative BCI if a high number of trials can be obtained per event. The following sections indicate relevant issues and challenges that would leverage the use of BCIs for healthy people in various situations.

### 6.1. Social Psychology

As several subjects shall be present in a cooperative BCI, it would be important to analyze people’s feelings, thoughts, and behaviors during a cooperative BCI experiment, which could be performed with people in the same place or online. In [39,40], the neural activity of different brains during daily life interactions is measured simultaneously by using EEG in order to understand the neural processes generating and generated by social cooperation or competition. Their results suggest that EEG hyperscannings could be used to investigate experimental paradigms where the knowledge of the simultaneous interactions between the subjects has a value. In a cooperative BCI, it would be possible to create paradigms replicating social situations, and the BCI feedback could provide key insights on human behavior. Cooperative BCI could be a tool to bridge the gap between cognitive neuroscience and social psychology. Furthermore, as motivation and reward have an impact on ERP characteristics, the cooperative aspect can be combined with a competitive approach where a feedback could indicate who is the main contributor of the command. Moreover, it may be easier to accept an error in a cooperative BCI system because the cause of the error can be attributed to other subjects.

### 6.2. Entertainment and Education

As described in the first section, a large number of subjects would increase the performance of a cooperative BCI. Typical environments where people are involved as a group in a simultaneous shared task can be classrooms and theaters. In a classroom, a cooperative BCI setting could provide a precise feedback to the teacher about the current mental state of the whole classroom (fatigue, difficulty of the class, …). In a theater, a cooperative BCI could be used to interact in real-time with the performer by providing feedback via the analysis of *α*-activity or motor imagery responses.

While BCIs have been appealing for games [41,42], current BCI games stay at the prototype stage by considering well-established paradigms [43,44]: they do not offer the level of performance that is expected in terms of accuracy, and they propose no added values compared with other devices for healthy people. For non-video games, the concept of cooperative BCI has already been used in the Mattel Mindflex toy. The cooperative BCI approach is used as a part of the game, where the goal is to race through an obstacle course as fast as possible. To leverage the use of BCI in video games, cooperative BCI systems are particularly promising due to the possible performance but also the inner principle of cooperative gaming. Cooperative games have a long history in the video game industry, e.g., two players co-op mode with Double Dragon (1987, Taito corporation, Shibuya, Tokyo, Japan), and it has continued to gain popularity over the years, e.g., having up to four players with Diablo 3 (2012, Blizzard Entertainment, Irvine, CA, USA). The increase of games with a cooperative option is mainly due to the development of the internet and new controllers. Among the different features present in a cooperative game, the idea of resource sharing between subjects is brought into consideration.

In the framework of cooperative BCIs based on ERP detection, a resource shared between subjects can be the attention capability [45,46]. The strength of an ERP, and therefore its detection, depends on attentional capacity. Subjects should therefore master their attention to enable ERP detection. In video games, quick time event (QTE) is a method of context-sensitive gameplay where a player performs an action after the appearance of a visual or auditory stimulus. The acronym QTE is attributed to Yu Suzuki, director of the game Shenmue, Sega, 1999. QTEs are often used during cutscene sequences to keep the attention of the player and to propose specific contextual gameplay (e.g., Resident Evil 4, Capcom, 2005). QTEs are typically the kind of event that would evoke an ERP and would enable the knowledge of the stimulus onset for ERP detection (the gameplay is not self-paced). Therefore, QTEs are the best type of event that could be successfully used in a cooperative BCI based on ERP detection. In addition, an ERP such as the P300 can be detected in a low frequency band (under 10 Hz), avoiding jitter issues in online games due to the lag.

Cooperative BCIs are relevant in multiplayer online video games where players work as a team to achieve a particular goal. In such cases a group of players cooperate as a collective to complete a quest (a raid). For instance, raid groups can have from 5 up to 40 people doing the same global task in World of Warcraft (2004, Blizzard Entertainment). In this context, the activation of events in the game could be achieved only if a particular mental state can be found across all subjects. In this situation, it would be easier to have access to a large pool of users aiming at achieving the same task. A recent trend in video games involves motion gaming, e.g., Wii fit (Nintendo), Kinect (Microsoft), Playstation Move (Sony). Though non-invasive detection of particular human motion is unreliable in comparison with camera or sensors placed on the body, the combination of brain responses corresponding to particular movement could be efficiently used. For instance, in a video game based on dance, several players would perform synchronously a series of movements from which one can detect the combination of signals coming from each subject. The activation of the command would depend not only on the performance of the subject but also on the performance of the whole group.

### 6.3. Materials and Technical Issues

The cost of a BCI for a single person can still be expensive and could be an obstacle for the development of cooperative BCIs. A cooperative BCI system should ideally require at least five people to provide reliable results in the case of ERP-based BCI. For instance, we have shown that it is possible to achieve a mean AUC of 0.939 with four electrodes common to five subjects. Therefore, each subject should wear an EEG cap and have a personal amplifier (if the subjects are at the same location, and if the amplifier can cater for different ground and reference configurations, it would be possible to share the same amplifier). Further investigations should be carried out at the economic level to determine if it is more likely to find customers willing to buy an expensive BCI product (with many electrodes) or customers who wish to pay for a cheaper product (with less electrodes) that would require several people to perform efficiently. In addition to hardware issues for setting a cooperative BCI, BCI frameworks should embed features for transferring signals and decisions online. These features are already available in current BCI frameworks, e.g., BCI2000, OpenViBE, MATLAB+Simulink [47,48], facilitating the development of cooperative BCIs. With cooperative BCIs for healthy people, it would be more efficient to have several users with relatively cheap systems with few electrodes than to have a single user with a very expensive system, e.g., with 32 channels. By considering the EEG signals from different users, a remaining issue is the synchronization of the different EEG signals to the different visual stimuli to avoid jitter effects.

## 7. Conclusions

A classification of BCI according to the number of BCI paradigms and the number of subjects involved has been proposed: the classical BCI involving one subject with one paradigm is extended to several subjects with one paradigm (cooperative), to one subject with several paradigms (hybrid), or several subjects and several paradigms (coop-hybrid). Since the early days of ERP analysis, trials have been averaged both over time and across subjects to increase the signal-to-noise ratio and to determine key findings in cognitive neuroscience. We have shown that combining trials across subjects could be of great use in particular BCI applications, as it has been achieved over time in ERP-based BCI; an analogy is from sequential process to parallel process. The best combination method for combining decisions across subjects is the mean, and while it is better to consider a larger number of subjects than a lot of electrodes for single-event detection, several applications could benefit from a configuration with multiple subjects, and a moderate number of electrodes. Cooperative BCIs represent a fundamental new approach compared with current BCI, because the user does not work independently but interacts with other people having the same goal. Moreover, the performance does not depend on a single user but on the group of users, hence to some extent limiting the drawback related to BCI illiteracy: each subject has a contribution to the overall performance even if this contribution is slight.
